# Viral MicroRNAs Repress the Cholesterol Pathway, and 25-Hydroxycholesterol Inhibits Infection

**DOI:** 10.1128/mBio.00576-17

**Published:** 2017-07-11

**Authors:** Anna K. P. Serquiña, Diane M. Kambach, Ontara Sarker, Joseph M. Ziegelbauer

**Affiliations:** a HIV and AIDS Malignancy Branch, National Cancer Institute, National Institutes of Health, Bethesda, Maryland, USA; b Radiation Oncology Branch, National Cancer Institute, National Institutes of Health, Bethesda, Maryland, USA; University of Michigan—Ann Arbor

**Keywords:** Kaposi's sarcoma-associated herpesvirus, cholesterol, human herpesviruses, microRNA

## Abstract

From various screens, we found that Kaposi’s sarcoma-associated herpesvirus (KSHV) viral microRNAs (miRNAs) target several enzymes in the mevalonate/cholesterol pathway. 3-Hydroxy-3-methylglutaryl-coenzyme A (CoA) synthase 1 (HMGCS1), 3-hydroxy-3-methylglutaryl-CoA reductase (HMGCR [a rate-limiting step in the mevalonate pathway]), and farnesyl-diphosphate farnesyltransferase 1 (FDFT1 [a committed step in the cholesterol branch]) are repressed by multiple KSHV miRNAs. Transfection of viral miRNA mimics in primary endothelial cells (human umbilical vein endothelial cells [HUVECs]) is sufficient to reduce intracellular cholesterol levels; however, small interfering RNAs (siRNAs) targeting only HMGCS1 did not reduce cholesterol levels. This suggests that multiple targets are needed to perturb this tightly regulated pathway. We also report here that cholesterol levels were decreased in *de novo*-infected HUVECs after 7 days. This reduction is at least partially due to viral miRNAs, since the mutant form of KSHV lacking 10 of the 12 miRNA genes had increased cholesterol compared to wild-type infections. We hypothesized that KSHV is downregulating cholesterol to suppress the antiviral response by a modified form of cholesterol, 25-hydroxycholesterol (25HC). We found that the cholesterol 25-hydroxylase (CH25H) gene, which is responsible for generating 25HC, had increased expression in *de novo*-infected HUVECs but was strongly suppressed in long-term latently infected cell lines. We found that 25HC inhibits KSHV infection when added exogenously prior to *de novo* infection. In conclusion, we found that multiple KSHV viral miRNAs target enzymes in the mevalonate pathway to modulate cholesterol in infected cells during latency. This repression of cholesterol levels could potentially be beneficial to viral infection by decreasing the levels of 25HC.

## IMPORTANCE

A subset of viruses express unique microRNAs (miRNAs), which act like cellular miRNAs to generally repress host gene expression. A cancer virus, Kaposi’s sarcoma-associated herpesvirus (KSHV, or human herpesvirus 8 [HHV-8]), encodes multiple miRNAs that repress gene expression of multiple enzymes that are important for cholesterol synthesis. In cells with these viral miRNAs or with natural infection, cholesterol levels are reduced, indicating these viral miRNAs decrease cholesterol levels. A modified form of cholesterol, 25-hydroxycholesterol, is generated directly from cholesterol. Addition of 25-hydroxycholesterol to primary cells inhibited KSHV infection of cells, suggesting that viral miRNAs may decrease cholesterol levels to decrease the concentration of 25-hydroxycholesterol and to promote infection. These results suggest a new virus-host relationship and indicate a previously unidentified viral strategy to lower cholesterol levels.

## INTRODUCTION

Various microRNAs (miRNAs) regulate many cellular processes by stalling translation of mRNA and by triggering mRNA degradation. Some viruses also utilize their own noncoding RNAs, such as viral miRNAs, to coopt cellular processes while avoiding detection by the immune system. Studying the interface of virus-host interactions at the level of viral miRNA modulation of host processes allows us to gain insights into pathways that can potentially be new therapeutic targets.

Kaposi’s sarcoma-associated herpesvirus (KSHV, or human herpesvirus 8 [HHV-8]) is one such virus that encodes its own suite of viral miRNAs. Previous reports have shown that KSHV viral miRNAs modulate processes involved in angiogenesis, inflammation, and immune evasion (1–4). KSHV is the causative agent of Kaposi’s sarcoma, the second most common cause of cancer in HIV-1 patients (5). KSHV infection also manifests clinically as pulmonary effusion lymphoma (PEL) and multicentric Castleman’s disease (MCD). KSHV latent infection has been shown to cause metabolic changes seen in cancer cells, such as the Warburg effect (increased glycolysis) (6), increased lipid synthesis (7), and increased glutaminolysis (8). Since HIV-1 patients are now living longer and having a higher likelihood of developing cancer, KSHV pathogenesis and its contribution toward oncogenesis remain an important topic of study.

**FIG 1:**
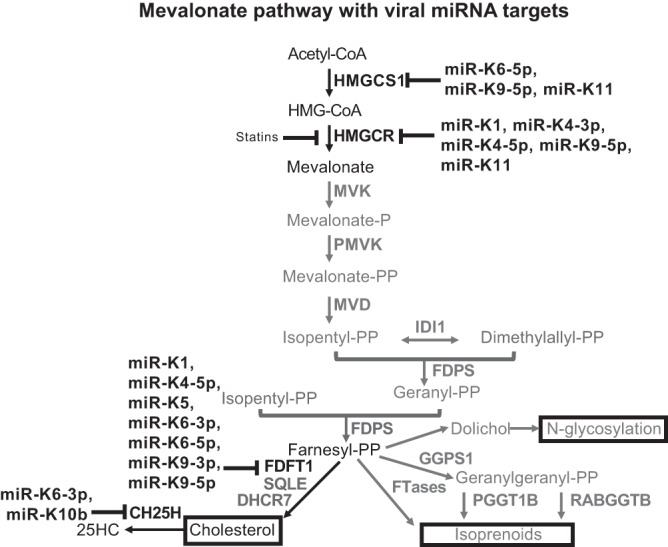
Schematic diagram of the mevalonate pathway with viral miRNA targets. Individual miRNAs that repress gene expression are shown.

Our previous screen for proteins repressed in the presence of KSHV miRNAs revealed that 3-hydroxy-3-methylglutaryl-coenzyme A (CoA) synthase 1 (HMGCS1), an enzyme of the mevalonate/cholesterol pathway, was one of the proteins most repressed by KSHV miRNAs (1). This is consistent with increasing evidence that different viruses perturb the homeostasis of the mevalonate/cholesterol pathway during infection. The mevalonate pathway (Fig. 1) is essential for cellular metabolism, particularly in *de novo* cholesterol biosynthesis, which is required for the maintenance of cellular membranes, with cholesterol a precursor of steroid hormones. This pathway is also responsible for synthesizing isoprenoids, which are used to tag specific proteins (geranylation or farnesylation) for membrane localization, and dolichol for N-glycosylation.

Other metabolites of the mevalonate pathway are also important for viral infection. For instance, geranyl-geranylation is required by the hepatitis C virus (HCV) to allow the viral protein NS5A to bind to the viral cofactor FBL2. Consequently, treatment with statins, which inhibit the mevalonate pathway, also blocked HCV replication (9; reviewed in reference 10). In Epstein-Barr virus (EBV)-infected lymphoma cell lines, simvastatin induced apoptosis by interfering with the localization and activity of EBV latent membrane protein 1 (LMP-1) (11). Cholesterol also gives rise to oxidized derivatives called oxysterols that may act as signaling molecules. One of these, 25-hydroxycholesterol (25HC), has recently been described as antiviral against a broad range of viruses (4, 12). Additionally, 25HC was the only oxysterol that was secreted in response to murine cytomegalovirus (MCMV) infection or interferon (IFN) treatment of murine macrophages (4).

While we have identified and validated HMGCS1 as a KSHV miRNA target, our present work describes our finding that KSHV viral miRNAs target additional enzymes in this pathway and that the same viral miRNAs repress gene expression of successive enzymes in the same pathway. We also explore whether KSHV perturbs cholesterol levels in *de novo* latent infection. Finally, we investigate how the virus may benefit from repressing the mevalonate/cholesterol pathway in latent infections.

## RESULTS

### Viral miRNA mimics suppress HMGCS1 protein expression.

**FIG 2:**
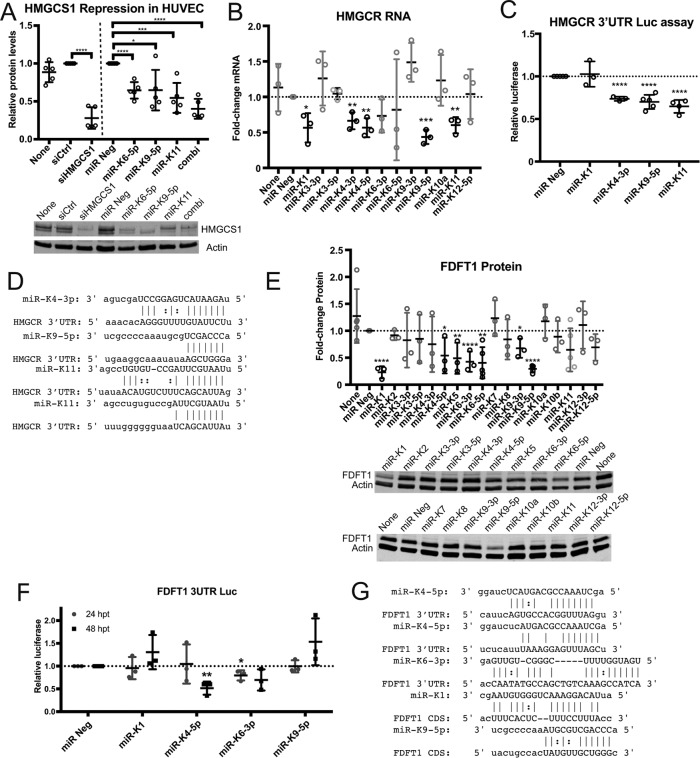
KSHV viral miRNAs target several enzymes in the mevalonate pathway. (A) HUVECs were transfected with either 30 nM siRNA or miRNA mimics. Total protein lysates were harvested at 48 h posttransfection (hpt) for immunoblotting with HMGCS1 and actin. combi, combination of 3 miRNA mimics transfected (*n =* 5). (B) HUVECs were transfected with KSHV viral miRNA mimics, and HMGCR gene expression was measured using RT-qPCR (*n =* 3). (C) Luciferase (Luc) reporter assay with full-length 3′ UTR of HMGCR cloned into a reporter plasmid (*Renilla* luciferase) and cotransfected with miRNA mimics in HEK-293 cells. Luciferase signal was normalized to an internal luciferase control (firefly luciferase), luciferase empty vector control, and negative-control miRNA (miR Neg) (*n =* 4). (D) Predicted miRNA target sites in the HMGCR 3′ UTR were identified using miRanda software. (E) HUVECs were transfected with KSHV viral miRNA mimics, and total protein lysates were harvested at 48 hpt for immunoblotting with anti-FDFT1 and antiactin (*n =* 3). (F) Luciferase reporter assay with full-length 3′ UTR of FDFT1 was performed as described above (*n =* 3). (G) Predicted viral miRNA target sites in FDFT1 3′ UTR and coding DNA sequence (CDS) were identified using miRanda software. For graphs, each data point represents a biological replicate, with error bars shown as means ± standard deviation (SD). Student’s *t* test was performed to assess statistical significance: *, *P* < 0.05; **, *P* < 0.01; ***, *P* < 0.001; ****, *P* < 0.0001.

To demonstrate that viral miRNAs can suppress HMGCS1 protein levels, we transfected human umbilical vein endothelial cells (HUVECs) with the viral miRNA mimics kshv-miR-K12-9-5p (miR-K9-5p), kshv-miR-K12-11-3p (miR-K11), and kshv-miR-K12-6-5p (miR-K6-5p). All three individual miRNA mimics resulted in repression of HMGCS1 protein expression by ~35% (Fig. 2A). Interestingly, we observed the strongest repression when three KSHV miRNAs were transfected in combination (~60%).

### Validation of other targets in the mevalonate pathway.

Aside from HMGCS1, we investigated whether KSHV viral miRNAs modulate other enzymes in the mevalonate pathway. Based on previous expression data with KSHV infection or transfection of viral miRNA mimics (13), we explored several other human genes of the mevalonate/cholesterol pathway that were repressed in primary endothelial cells with KSHV infection or transfection of KSHV miRNA mimics compared to control cells (Table 1). Additionally, we explored a published data set (PAR-CLIP [photoactivatable ribonucleoside enhanced cross-linking and immunoprecipitation]) of binding sites of miRNA-containing complexes from KSHV PEL cell lines with their corresponding viral miRNA binding sites (14) and found several candidate genes of the mevalonate pathway that may be targeted by KSHV viral miRNAs (see Table S1 in the supplemental material). Using this information, we sought to investigate repression of specific genes by specific viral miRNAs.

10.1128/mBio.00576-17.4 TABLE S1  PAR-CLIP (photoactivatable ribonucleoside enhanced cross-linking and immunoprecipitation) identified binding sites of viral miRNAs on target genes of the mevalonate/cholesterol pathway (14). Download TABLE S1, DOCX file, 0.02 MB. Copyright © 2017 Serquiña et al. 2017 Serquiña et al. This content is distributed under the terms of the Creative Commons Attribution 4.0 International license.

**TABLE 1:** Gene expression profile for the mevalonate/cholesterol pathway in primary endothelial cells

Gene product	Expression profile for^a^:	
	+KSHV (48 hpi)	+KSHV miRNAs (30 hpt)
HMGCS1	−0.656	−0.270
HMGCR	−0.283	−0.377
MVK	ND	ND
PMVK	−0.005	−0.175
MVD	ND	ND
IDI1	−0.235	−0.168
FDPS	−0.165	0.046
FDFT1	−0.390	−0.110
GGPS1	ND	ND
DHCR7	−0.215	0.004
PGGT1B	0.121	−0.033
RABGGTB	ND	ND
CH25H	ND	ND

^a^ Shown are the gene expression profiles in primary endothelial cells that were either *de novo* infected (+KSHV) and assayed at 48 hpi or transiently transfected with KSHV viral miRNA mimics (+KSHV miRNAs) and assayed at 30 hpt. Gene expression relative to the appropriate control (mock infected or miRNA negative control transfected) is shown in the log_2_ scale. ND, not detected.

3-Hydroxy-3-methylglutaryl-CoA reductase (HMGCR) catalyzes the rate-limiting step in the mevalonate pathway and is competitively inhibited by statins, which are used clinically to lower plasma cholesterol levels. To determine whether viral miRNAs can target HMGCR, we transfected viral miRNA mimics individually in primary endothelial cells and assayed for HMGCR mRNA expression using quantitative reverse transcription-PCR (RT-qPCR) (Fig. 2B). We found that several viral miRNAs significantly suppress HMGCR mRNA levels—namely, kshv-miR-K12-1-5p (miR-K1), kshv-miR-K12-4-3p (miR-K4-3p), kshv-miR-K12-4-5p (miR-K4-5p), miR-K9-5p, and miR-K11. To confirm that these viral miRNAs directly target the 3′ untranscribed region (UTR) of HMGCR, we performed a luciferase reporter assay wherein the 3′ UTR of HMGCR was cloned into the reporter plasmid and the viral miRNAs were cotransfected with the reporter plasmid. With this assay, we found that only miR-K4-3p, miR-K9-5p, and miR-K11 caused a decrease in the luciferase signal (Fig. 2C), implying that they can directly bind to the 3′ UTR of HMGCR and downregulate HMGCR mRNA expression. In contrast, miR-K1 may target the HMGCR mRNA outside the 3′ UTR, such as in the coding sequence. Data from cross-linking and immunoprecipitation experiments support a miR-K1 site inside the coding sequence (Table S1). Using the miRNA target prediction program miRanda (15), we performed a search for seed matches in the 3′ UTR of the HMGCR mRNA and found one seed match for miR-K4-3p and miR-K9-5p and two seed matches for miR-K11 within the 3′ UTR of HMGCR (Fig. 2D). It is notable that miR-K9-5p and miR-K11 also target the 3′ UTR of HMGCS1 (1), the enzyme immediately upstream of HMGCR in the mevalonate pathway.

The farnesyl-diphosphate farnesyltransferase 1 (FDFT1) gene encodes the enzyme squalene synthase (SQS) which catalyzes the first specific step in the cholesterol synthesis branch of the mevalonate pathway. To determine whether any KSHV miRNAs can target FDFT1 gene expression, we performed a similar screen to that described above and assayed for FDFT1 protein using immunoblotting. We found that several viral miRNAs suppressed FDFT1 protein expression (Fig. 2E), including miR-K1, miR-K4-5p, miR-K5, miR-K6-3p, miR-K6-5p, miR-K9-3p, and miR-K9-5p. We proceeded to confirm whether these miRNAs directly target the 3′ UTR of FDFT1. We found that only miR-K6-3p (at 24 h posttransfection [hpt] [Fig. 2F]) and miR-K4-5p (at 48 hpt [Fig. 2F]) caused decreased luciferase signal, implying that they target the 3′ UTR of FDFT1 directly. We did not observe repression of the FDFT1 3′ UTR luciferase reporter with miR-K9-5p. Using miRanda, we found potential binding sites for miR-K4-5p (Fig. 2G) and for miR-K6-3p in the 3′ UTR of FDFT1. Upon further analysis, we found a potential binding site for miR-K1 and miR-K9-5p in the coding DNA sequence of FDFT1 (Fig. 2G). The miR-K1 site was also found in previous cross-linking and immunoprecipitation experiments [Table S1], which may explain why the 3′ UTR reporter assay did not reflect any changes when miR-K1 or miR-K9-5p was cotransfected. Together, these results suggest that some KSHV miRNAs are targeting sequences in the 3′ UTR and some are targeting sequences in the coding sequence of FDFT1 to repress expression of FDFT1.

### Effect of viral miRNAs on cholesterol.

**FIG 3:**
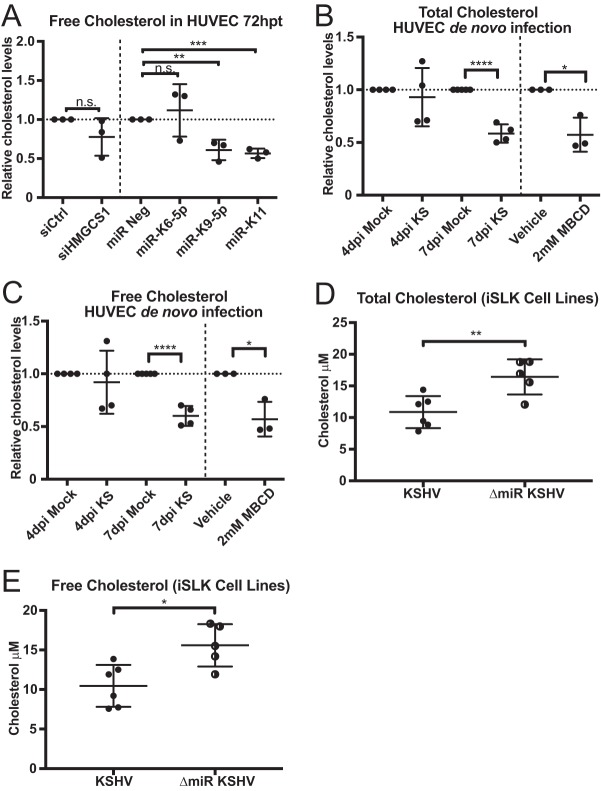
KSHV latent infection perturbs cellular cholesterol levels. (A) HUVECs were transfected with either 30 nM siRNA or miRNA mimics. The graph shows data points for experiments normalized to negative control (siCtrl, miR Neg) (*n =* 3). (B and C) Total (B) and free (C) cholesterol assays on HUVECs infected with BCBL1-derived virus. Cholesterol samples were extracted and assayed as described above. Data points are normalized to the uninfected control (Mock). As a positive control, HUVECs were treated with 2 mM MBCD (cholesterol inhibitor) for 2 h prior to harvest (*n =* 4). (D and E) Total (D) and free (E) cholesterol assays on iSLK cell lines infected with either wild-type KSHV or mutant virus lacking the miRNA cluster (ΔmiR KSHV). Cells were harvested and assayed as described above (*n =* 5 to 6). Statistical significance: n.s., not significant; *, *P* < 0.05; **, *P* < 0.01; ***, *P* < 0.001; ****, *P* < 0.0001.

Since the mevalonate pathway results in cholesterol synthesis, we explored whether viral miRNA-dependent repression of gene expression of pathway genes resulted in decreased intracellular cholesterol levels. We transfected HUVECs with viral miRNA mimics and then extracted and measured cholesterol levels. Our results showed that only miR-K9-5p and miR-K11 (both target HMGCS1 and HMGCR 3′ UTR) caused decreased cholesterol synthesis (*P* = 0.0067 and *P* = 0.0003, respectively), while miR-K6-5p (which does not target the HMGCS1 3′ UTR) had no significant effect (Fig. 3A). Interestingly, depletion of HMGCS1 alone with siRNA did not significantly alter the cholesterol levels. Also, only viral miRNAs that target HMGCS1 and HMGCR 3′ UTRs perturbed cholesterol levels (Fig. 3A). This suggests that miRNA-mediated repression of multiple genes in the pathway is necessary to lower intracellular cholesterol levels.

We also wanted to determine the effect of KSHV *de novo* infection on cholesterol levels. We therefore infected HUVECs with KSHV and harvested cells at different time points. We measured total and free cholesterol and found that both levels of cholesterol were decreased at 7 days postinfection (dpi) (Fig. 3B and C) but not at 4 dpi. Treatment with methyl-β-cyclodextrin (MBCD) was used as a positive control for cholesterol depletion.

To determine the specific effect of viral miRNAs on cholesterol levels during KSHV infection, we compared cholesterol extracted from iSLK cells infected with either wild-type (WT) KSHV or a mutant virus (ΔmiR KSHV) with the miRNA cluster deleted (16). Interestingly, we observed that iSLK cells infected with ΔmiR KSHV virus had increased total and free cholesterol (Fig. 3D and E) compared to iSLK cells infected with WT virus. This suggests that KSHV viral miRNAs facilitate suppression of cholesterol levels and that removal of the viral miRNA cluster abrogates this phenomenon.

### CH25H and 25HC as an antiviral response.

**FIG 4:**
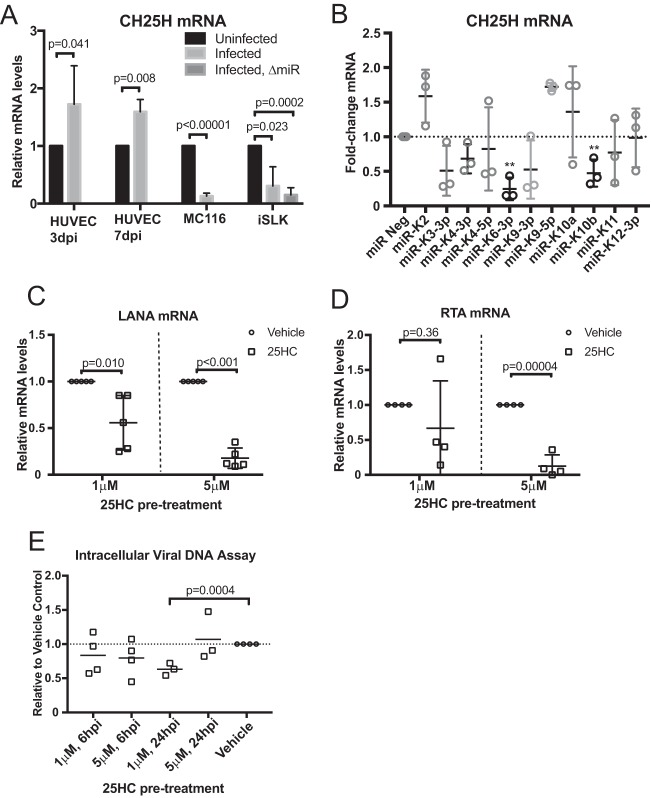
CH25H and 25HC during KSHV infection. (A) CH25H gene expression measured by RT-qPCR assay from *de novo*-infected HUVECs, from the B cell line MC116, and from iSLK cells infected with either wild-type or ΔmiR mutant KSHV. As an uninfected control for iSLK, an SLK cell line was assayed (*n =* 3). (B) HUVECs were transfected with a KSHV viral miRNA mimics, and CH25H gene expression was measured using RT-qPCR (*n =* 3). (C and D) HUVECs were pretreated with 25HC or vehicle only (ethanol) at 2 h prior to infection with BCBL1-derived virus supernatant. mRNA samples were harvested at 2 dpi. LANA (*n =* 5) and RTA (*n =* 4) mRNA expression was measured using the RT-qPCR assay and normalized to β-actin. (E) HUVEC pretreatment and infection as described above. At indicated time points, cells were washed and trypsinized to remove virions adhering to cell surface. DNA was extracted, and LANA DNA was measured using qPCR and normalized to human DNA (*n =* 4). **, *P* < 0.01.

With the results that showed viral miRNAs repressed mevalonate gene expression and reduced cholesterol levels, we next investigated how the virus may benefit from a repression of cholesterol levels. We hypothesized that a product of the mevalonate pathway may possess antiviral activities and repression of the mevalonate pathway may decrease the levels of the antiviral molecule. One potential antiviral factor of interest was cholesterol 25-hydroxylase (CH25H), which is the enzyme that mainly converts cholesterol to 25-hydroxycholesterol (25HC). Notably, CH25H expression has also been reported to restrict replication of murine gammaherpesvirus 68 (MHV68), which is closely related to KSHV (12, 17). In the context of MHV68, the loss of CH25H in macrophages (CH25H^−/−^) resulted in increased replication and transcription activator (RTA) mRNA expression and increased viral DNA synthesis (17). CH25H expression is induced in MHV68 *de novo* infection of primary macrophages shortly after infection. We hypothesized that CH25H and its product, 25HC, are part of the innate immune response against KSHV *de novo* infection and that KSHV viral miRNAs downregulate the mevalonate pathway to decrease the amount of cholesterol, which is the substrate of CH25H. Reduced levels of cholesterol may also reduce the levels of 25HC. We found that CH25H is consistently increased during *de novo* infection of HUVECs (primary cells) but repressed in latently infected cell lines MC116.219 and iSLK (Fig. 4A). CH25H expression was also repressed by KSHV viral miRNAs miR-K6-3p and miR-K10b (Fig. 4B; *P* = 0.0013 and 0.0098, respectively). This suggests that host responses may increase CH25H expression, but repression of upstream steps in the mevalonate pathway and repression of CH25H by KSHV miRNAs may function to counteract the antiviral effects of CH25H’s production of 25HC.

### CH25H is not interferon inducible in human endothelial cells.

The CH25H gene has previously been reported to be an interferon (IFN)-responsive gene (IFN-α and -γ in reference 12 and IFN-β in reference 18) in murine bone marrow-derived macrophages (BMDMs) (12). We tested whether CH25H is also IFN inducible in primary human endothelial cells but found that it was not (IFN-β and -γ; see Fig. S1C and D in the supplemental material), while IRF1 and IFN-stimulated gene 15 (ISG15) were induced (Fig. S1A and B). Our results correlate with earlier work (19), which showed that the CH25H gene was not an IFN-stimulated gene in human liver cell lines.

10.1128/mBio.00576-17.1 FIG S1  CH25H gene expression in HUVECs is not interferon inducible. HUVECs were treated with either 5 ng/ml IFN-β or 5 ng/ml IFN-γ, and RNA samples were collected at the indicated time points. To confirm interferon induction, gene expression levels for (A) IRF1 and (B) ISG15 were measured by RT-qPCR and normalized to β-actin mRNA expression. CH25H gene expression was measured by RT-qPCR assay with (C) IFN-γ and (D) IFN-β treatment. Download FIG S1, EPS file, 1.6 MB. Copyright © 2017 Serquiña et al. 2017 Serquiña et al. This content is distributed under the terms of the Creative Commons Attribution 4.0 International license.

### 25HC represses KSHV *de novo* infection.

To determine whether 25HC is antiviral against KSHV, we pretreated HUVECs with 25HC for 2 h prior to *de novo* infection. Two days postinfection (dpi), we investigated viral infection efficiency by measuring viral gene expression (normalized to cellular β-actin expression) and found that latency-associated nuclear antigen (LANA) mRNA expression was decreased in a dose-dependent fashion with 25HC pretreatment (Fig. 4C). We also assayed for viral replication and transcription activator (RTA), initially expressed during *de novo* infection (20), and found that RTA mRNA expression was also significantly decreased with 5 µM 25HC treatment (Fig. 4D). We also tested the effect of adding exogenous 25HC after *de novo* infection but did not observe a significant difference (see Fig. S2A and B in the supplemental material). We wanted to investigate the mechanism of how 25HC exerts its antiviral effect on the KSHV life cycle. To address this, we assayed for viral entry with 25HC pretreatment of HUVECs using quantitative PCR for KSHV DNA (20) (Fig. 4E). We did not observe a strong repression of entry after 6 h of infection, when entry inhibition would be expected, as viral DNA increased until 4 h after KSHV infection (21). These results taken together suggested that 25HC inhibits KSHV infection at a postentry step (similar to observations from 25HC and MCMV [4]) and that 25HC may disrupt infection only at the time of new infection, since treatment with 25HC after infection did not repress KSHV gene expression. We investigated if 25HC could inhibit LANA promoter activity by using three different LANA promoter regions fused to a luciferase reporter. However, 25HC did not inhibit LANA promoter activity in these assays (see Fig. S3C in the supplemental material). Note that these assays test cellular factors controlling LANA promoter activity, and potential effects of 25HC on viral proteins interacting with the LANA promoter were not measured in this specific assay. Other tests investigated whether delivery of viral DNA to the nucleus was inhibited by 25HC, but no significant inhibition was observed (Fig. S3D). Together, these results suggest that 25HC likely inhibits a step between viral DNA delivery to the nucleus and stable expression of viral genes.

10.1128/mBio.00576-17.2 FIG S2  Gene expression of SREBP2, SCAP, and FASN. Samples from Fig. 4A (HUVECs) were measured for gene expression of (A) SREBP2, (B) SCAP, and (C) FASN using the RT-qPCR assay. Download FIG S2, EPS file, 1.3 MB. Copyright © 2017 Serquiña et al. 2017 Serquiña et al. This content is distributed under the terms of the Creative Commons Attribution 4.0 International license.

10.1128/mBio.00576-17.3 FIG S3  25HC treatment and viral transcription. HUVECs were infected with BCBL1-derived virus supernatant. After 6 h, virus was washed off and 25HC or vehicle (ethanol) was added to growth media. mRNA samples were harvested at 2 dpi. (A and B) LANA (A) and RTA (B) mRNA expression was measured using RT-qPCR and normalized to β-actin mRNA expression. (C) Three different LANA promoter regions were cloned upstream of luciferase reporters. Cells were pretreated with 25HC before transfection with the luciferase reporters. (D) Nuclei were isolated to test for nuclear delivery of viral DNA using qPCR and normalized to human DNA. Download FIG S3, EPS file, 1.6 MB. Copyright © 2017 Serquiña et al. 2017 Serquiña et al. This content is distributed under the terms of the Creative Commons Attribution 4.0 International license.

**FIG 5:**
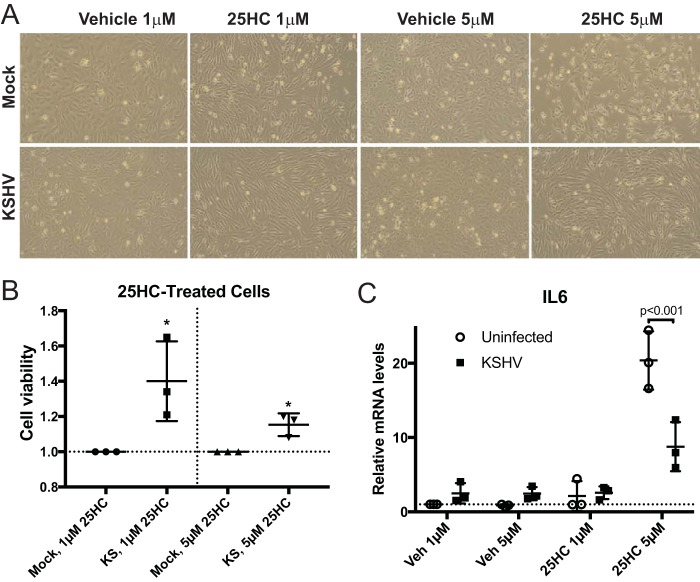
25HC’s effects on viability and IL-6 expression. (A) Micrographs of HUVECs pretreated with 25HC and infected as described in the legend to Fig. 4. (B) HUVECs were treated with 25HC and infected with KSHV, with cell viability measured by WST-1 assays (*n =* 3). (C) RNA samples used in Fig. 4C and D were also used to measure IL-6 mRNA expression (*n =* 3). Veh, vehicle. *, *P* < 0.05.

In addition to a defect in establishing *de novo* infection in HUVECs, we noticed that 25HC treatment caused an elongated morphology in the cells (Fig. 5A; 25HC treated versus vehicle treated in both mock-infected and infected cells). Spindle cells, which are the histopathologic hallmark in Kaposi’s sarcoma lesions, also have this marked abnormal cell shape. Furthermore, we also noted more spaces in the monolayer of uninfected cells treated with 25HC compared to infected cells treated with 25HC. This observation was measured using a standard cell viability assay that measures metabolically active cells, which confirmed the observation that 25HC treatment reduced viability of uninfected cells more than that of infected cells (Fig. 5B). This suggests that KSHV-infected cells may survive the 25HC treatment better than uninfected cells since infected cells may have less 25HC than uninfected cells, due to the decreased cholesterol levels in infected cells. We also investigated other consequences of 25HC treatment and found that 25HC treatment dramatically increased interleukin-6 (IL-6) expression in uninfected cells (20-fold), but the activation of IL-6 by 25HC was significantly repressed in KSHV-infected cells (Fig. 5C). We note that we observed a 4-fold increase in IL-6 expression in vehicle-treated cells, comparing infected cells to uninfected cells, which has been previously observed by other groups. However, the decreased activation of IL-6 by 25HC in infected cells compared to uninfected cells correlates with the increased viability in the infected cells in the presence of 25HC (Fig. 5A and B), suggesting that higher levels of IL-6 activation could be detrimental to cell viability and inhibitory to *de novo* infection (Fig. 4C and D) (see Discussion).

## DISCUSSION

We have demonstrated that multiple KSHV viral miRNAs modulate several enzymes in the mevalonate/cholesterol pathway (Fig. 1). There is redundancy in this repression, such that the same miRNAs target different enzymes in the same pathway. In addition, we saw the best repression of HMGCS1 when multiple KSHV miRNAs were introduced into cells. We have also shown that the amounts of free and total cholesterol decreased with infection in primary endothelial cells after 7 days. Viral miRNAs are at least partially responsible for this cholesterol suppression, since deletion of the miRNA cluster resulted in increased cholesterol compared to wild-type infection.

Previous reports have demonstrated that both viral infections (including murine cytomegalovirus, herpes simplex virus 1, Semliki Forest virus, vaccinia virus, and adenovirus) and IFN-γ treatment of murine bone marrow-derived macrophages (BMDMs) downregulate genes in the mevalonate pathway (22). The authors concluded that this downregulation is due to a virus-induced interferon response.

Complementary to this, York et al. (23), demonstrated that the depletion of regulators of the mevalonate pathway, namely, sterol regulatory element-binding protein (SREBP) and SREBP cleavage-activating protein (SCAP), results in increased basal expression of IFN-β1 and an IFN-stimulated gene (ISG) signature in murine BMDMs. They have put forth the hypothesis that decreased flux of metabolites through the cholesterol arm of the mevalonate pathway facilitates the IFN response to viral infections.

While previous reports have shown that mevalonate/cholesterol downregulation is due to a virus-induced interferon response and that knockout of the master regulators of the pathway (SREBP and SCAP) facilitated an increased basal interferon signature, in the context of KSHV, this is not the same (Fig. S3). There is also no concomitant significant increase in fatty acid synthase (FASN) gene expression.

We postulate that KSHV downregulates the mevalonate/cholesterol pathway to prevent the formation of antiviral 25HC by the enzyme CH25H, which oxidizes cholesterol. This is based on work from other groups which showed that 25HC is broadly antiviral (4, 12) and can amplify inflammation signaling in response to viruses (24). Blanc et al. (4) estimated that intracellular levels of 25HC in macrophages were between 0.05 and 20 nM. Secreted concentrations of 25HC ranged from 100 to 600 nM. The same article reported 50% inhibitory concentration (IC_50_) values of exogenous 25HC ranging from 0.4 to 2 µM. Gold et al. (24) used 5 µM exogenous 25HC in their studies. Thus, our utilized concentrations of 25HC are in the range of previous articles. The antiviral mechanism of 25HC seems to vary depending on the virus and ranges from blocking fusion (Nipah virus, [12]) to blocking entry (HIV-1 and vesicular stomatitis virus [VSV] [12]), formation of double membrane vesicles needed for viral replication (HCV [25]), and glycosylation of membranes on progeny virions (Lassa virus [26]). CH25H has also been reported to have a nonenzymatic antiviral mechanism by disrupting viral protein dimerization in HCV (27). In agreement with an earlier report on MHV68 (17), we observed that CH25H mRNA is upregulated in *de novo*-infected primary endothelial cells but downregulated in the stably infected cell lines iSLK and MC116.219. This suggests that there may exist antiviral host mechanisms to upregulate CH25H upon *de novo* infection (Fig. 3A; 3 dpi) and viral miRNAs suppress multiple steps of the mevalonate pathway to suppress this antiviral response. Viral gene products may eventually suppress the upregulation of CH25H and long-term latency is established. We also note that in primary endothelial cells, a recent report (28) demonstrated that cholesterol can induce MCPIP1 expression and mediate cholesterol-induced DNA damage. We recently reported that MCPIP1 can also inhibit expression of the majority of KSHV microRNAs (29).

Since the data suggest that CH25H and 25HC increase as part of an initial antiviral response to KSHV, we then tested whether 25HC induces an antiviral state in HUVECs prior to infection. Indeed, pretreatment of HUVECs with 25HC resulted in dose-dependent decrease in viral infection. While 25HC does not significantly affect viral entry at early time points, it is possible that 25HC may exert its effect at postentry steps of the viral life cycle. Others have demonstrated that 25HC induced retinoic inducible gene I (RIG-I) 9-fold (1) in the same primary endothelial cells used in our study. RIG-I signaling is known to be antiviral and has been specifically shown to inhibit KSHV viral gene expression (30). The specific mechanisms of how 25HC inhibits KSHV infection at postentry steps remain to be fully understood but may involve RIG-I signaling. In addition, we observed strong activation of human IL-6 by 25HC (Fig. 5). This is noteworthy because when IL-6 was added after HSV infection, viral titers were decreased in an IL-6 dose-dependent fashion (31). This suggests that high levels of IL6 may inhibit KSHV replication, similar to HSV-1 infection.

In contrast to studies on murine macrophages, we found that CH25H is not interferon inducible in human primary endothelial cells (Fig. S1). Our data are consistent with observations by Xiang et al. on human hepatic primary cells and cell lines in the context of HCV (19). It is conceivable that macrophages could be producing 25HC in response to virus-induced IFN signaling and that the secreted 25HC could then exert its effect on nearby cells.

We have described here how it may be advantageous to the virus to suppress the mevalonate pathway to establish latent infections. However, this repression of cholesterol synthesis may not be important during viral lytic reactivation. The lytic replication may require increased cholesterol for the formation of membranes required by progeny virions.

In summary, we demonstrated how KSHV viral miRNAs can downregulate the mevalonate/cholesterol pathway. We also described how a cholesterol derivative, 25-hydroxycholesterol, is antiviral against KSHV. Understanding the importance of the mevalonate/cholesterol pathway in viral infections may reveal new strategies for combating viral infections.

## MATERIALS AND METHODS

### Cell culture, reagents, and transfections.

Human umbilical vein endothelial cells (HUVECs) were obtained from Lonza and passaged in EGM2 medium (Lonza) for up to 5 passages, with passages 3 to 5 used for experiments. HUVECs were transfected using Dharmafect 1 following the manufacturer’s recommendations. The infected iSLK cell lines (WT and ΔmiR cluster deletion mutant virus) were kind gifts from Rolf Renne (16). They were maintained in Dulbecco’s modified Eagle’s medium (DMEM) with 10% fetal bovine serum (FBS) supplemented with hygromycin (1200 μg/ml), puromycin (1 μg/ml), and G418 (250 μg/ml). Lymphoma cell lines MC116 and MC116.219 were kind gifts from Ed Berger (32). Both were maintained in RPMI 1640 medium with 20% fetal bovine serum (FBS), and MC116.219 was supplemented with 10 μg/ml puromycin. LANA promoter luciferase plasmids were provided by Robert Yarchoan (33). ON-TARGETplus nontargeting control siRNA and ON-TARGETplus SMARTpool siRNA targeting HMGCS1 were obtained from Dharmacon/Thermo Fisher Scientific. KSHV miRNA mimics were obtained from Ambion. When transfecting the combination of miRNA mimics, 1/3 vol of each mimic was combined for a final concentration of 30 nM. 25HC (H1015; Sigma) was reconstituted in ethanol at 5 mM and stored in single-use aliquots at −20°C for no more than 4 weeks.

### Immunoblotting.

Total cell lysates were harvested with radioimmunoprecipitation assay (RIPA) buffer (Sigma) plus 1× Halt protease and phosphatase inhibitor cocktail (Thermo Scientific). Immunoblotting was performed using the following primary antibodies: anti-HMGCS1 rabbit polyclonal antibody (sc-33829; Santa Cruz Biotech), anti-squalene synthetase/FDFT1 rabbit polyclonal antibody (catalog no. GTX108885; GeneTex), and anti-β-actin mouse monoclonal antibody (AC-74; Sigma). Secondary antibodies were obtained from LI-COR. Immunoblots were imaged using an Odyssey Infrared Imaging System and quantified using Image Studio software (LI-COR). Signals from the protein of interest were normalized to β-actin levels and then expressed relative to the negative control.

### Luciferase assay.

Full-length 3′ UTRs of HMGCR and FDFT1 were cloned downstream of the *Renilla* luciferase reporter gene (Leidos). HEK-293 cells were reverse transfected with KSHV miRNA mimics and luciferase reporter vectors in triplicate. Luciferase assays were performed using the dual-luciferase reporter system (Promega) at 24 and 48 h posttransfection. Luminescence signal from *Renilla* luciferase was normalized to firefly luciferase signal (internal control) and to the empty vector (no 3′ UTR). Each data point represents an independent experiment (average of triplicate values).

### RT-qPCR.

mRNA was extracted using the RNeasy kit (Qiagen). Quantitative reverse transcription-PCR (RT-qPCR) was performed using 200 ng RNA and random primers with an Applied Biosystems high-capacity cDNA reverse transcription kit. SYBR green assays (FastStart universal SYBR green master mix; Roche) and TaqMan assays (TaqMan Universal PCR master mix, no AmpErase UNG; Applied Biosystems) were performed using the ABI StepOnePlus real-time PCR system (Applied Biosystems).

Relative mRNA levels were computed using the threshold cycle (ΔΔ*C*_*T*_) method with genes coding for β-actin or GAPDH (glyceraldehyde-3-phosphate dehydrogenase) as the reference genes.

### Cholesterol extraction and assay.

Cholesterol was extracted from cells using either the standard hexane-isopropanol method or a modified Bligh and Dyer method (34). The modified Bligh and Dyer method was carried out entirely with glass labware. Briefly, 0.5 million cells in duplicate were resuspended in 200 μl phosphate-buffered saline (PBS) and immediately frozen on dry ice and stored at −20°C or processed immediately. To the cell suspension, 700 μl of a 2:1 methanol-chloroform solution was added, and the tube was vortexed, followed by addition of 300 μl chloroform and a second round of vigorous vortexing. Finally, 250 μl of 1 M NaCl was added and mixed by vortexing. Samples were centrifuged at 3,000 × *g* for 15 min at 4°C, and the organic (bottom) phase was collected with a Pasteur pipette into a fresh glass test tube. Samples were air dried in a chemical hood for no more than 18 h. Cholesterol was resuspended in 80 μl of 5× Amplex buffer with periodic vortexing for 1 h, followed by dilution with an additional 320 μl water (to a final concentration of 1× buffer). The Amplex Red cholesterol assay (Molecular Probes) was carried out per the manufacturer’s instructions.

### Infections and viral entry assay.

*De novo* infections in HUVECs were carried out by diluting concentrated BCBL-1 supernatant in EGM2 medium at a multiplicity of infection (MOI) of 40, as determined by LANA copy number. Polybrene was added at 8 μg/ml. Virus supernatant were washed off after 6 h and replaced with fresh EGM2. HUVECs were refed every 2 days until harvest. The viral entry assay was performed as previously described (20).

### Data analysis and statistics.

Most graphs contain plots with each data point represented and also include the mean and standard deviations (SD). For testing of significance, *t* tests were used, with asterisks indicating various *P* values.
