# Supplementary material for: Viral MicroRNAs Repress the Cholesterol Pathway, and 25-Hydroxycholesterol Inhibits Infection
Source: mBio. 2017 Jul 11;8(4):e00576-17. doi: 10.1128/mBio.00576-17 (PMC5513709; doi:10.1128/mBio.00576-17)
Supplement: TABLE S1 [file mbo003173379st1.docx]

**Table S1.** PAR-CLIP (photoactivatable ribonucleoside-enhanced crosslinking and immunoprecipitation) identified binding sites of viral miRNAs on target genes of the mevalonate/cholesterol pathway (15).

| Gene | miRNA | LOCATION |
| --- | --- | --- |
| HMGCS1 | miR-K11 | 3’UTR |
| HMGCR | miR-K4-3p | intron |
| HMGCR | miR-K1 | CDS |
| HMGCR | miR-K4-3p | 3’UTR |
| MVK | miR-K9 | CDS |
| GGPS1 | miR-K6-3p | 3’UTR |
| IDI1 | miR-K10b | CDS |
| RABGGTB | miR-K5 | CDS |
| FDFT1 | miR-K12-3p | CDS |
